# A Rare Case of Painful Hand Swelling

**DOI:** 10.7759/cureus.29691

**Published:** 2022-09-28

**Authors:** Felwa A AlMarshad, Nehal Mahabbat, Ahmed Alkhalifah, Mohammed I Alhumaidan, Abdulaziz Jarman

**Affiliations:** 1 Plastic and Reconstructive Surgery Section, Department of Surgery, King Faisal Specialist Hospital and Research Centre, Riyadh, SAU; 2 Plastic Surgery, King Saud Bin Abdulaziz University for Health Sciences College of Medicine, Riyadh, SAU

**Keywords:** chronic wrist pain, rare anatomical variants, hand swelling, extensor indicis proprius, extensor digitorum brevis manus, s: magnetic resonance imaging

## Abstract

Many conditions are known to cause chronic wrist pain, one of which is accessory muscles which can be easily overlooked as the cause of pain. Here we present a case of chronic wrist pain in a 33-year-old male who presented with painful dorsal unilateral right-hand swelling associated with increased activity. This patient was referred to the radiology department and was found to have an accessory muscle along the dorsal aspect of the wrist consistent with the extensor digitorum brevis manus muscle. The report includes the clinical presentation, radiologic findings, and management.

## Introduction

A variety of accessory muscle variations in the hands had been described throughout the literature. These accessory muscles are discovered incidentally during surgeries, postpartum autopsy, or by using advanced imaging techniques [1-2]. These accessory muscles can be asymptomatic or can cause a variety of symptoms and can easily be confused as soft tissue tumors [3]. The extensor digitorum brevis manus (EDBM) is a striated skeletal muscle first described in 1758 by the Dutch anatomist Bernhard Albinus [3]. It is considered to be a normal variant with a prevalence of 2.5% found in true cadaveric autopsy [4]. Moreover, there is no association between EDBM presence and family history, gender, or ethnicity [4].

In the prenatal phase, the first indication of upper limb musculature is observed in the 7th week of development as condensation of the mesenchyme derived from dorsolateral cells of the somites [5]. The precursor muscle that gives rise to the forearm extensors differentiates into superficial, deep, and radial segments. The two most consistent theories found in the literature about the embryological origin of EDBM are: (1) EDBM arises from the deep portion of the forearm extensor precursor muscle [4], and (2) Failure in the proximal migration of the undifferentiated extensor precursor muscle representing an atavistic pattern [6-7].

Clinically, EDBM is one of the causes of what has been described as fourth compartment syndrome which is chronic dorsal wrist pain in the fourth compartment of the hand [8]. Therefore, listing EDBM as a possible differential diagnosis of chronic wrist pain can help hand surgeons to interpret its clinical implications accurately. Here we present a case of accessory muscle found along the dorsal aspect of the wrist consistent with extensor digitorum brevis manus muscle.

## Case presentation

**Figure 1:**
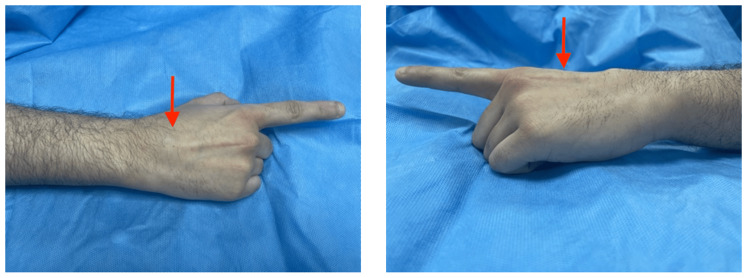
Images of the patient’s right hand Dorsal and lateral aspect, a swelling can be appreciated in the dorsum of the hand representing EDBM muscle (red arrows) EDBM: Extensor digitorum brevis manus muscle.

A 33-year-old male presented to the plastic surgery clinic complaining about a swelling in the dorsal aspect of his right hand that only became painful three months prior to his presentation. The swelling was noticed since his childhood and progressively increased in size. The pain was increasing with activities and was relieved with rest. Furthermore, the patient reported that the swelling resolves sometimes therefore our impression was Ganglion cyst and we planned for aspiration. However, the patient also reported that the swelling splits into two discrete swellings therefore we opted for magnetic resonance imaging (MRI) imaging. The patient did not report any other symptom such as numbness, weakness, or swelling on the contralateral hand. The patient did not have any past medical or surgical history. On examination, there was discrete swelling on the dorsal aspect of the right hand that became more prominent with dorsiflexion of the right hand and fingers (Figure 1). No discoloration or lymphadenopathy was found.

**Figure 2:**
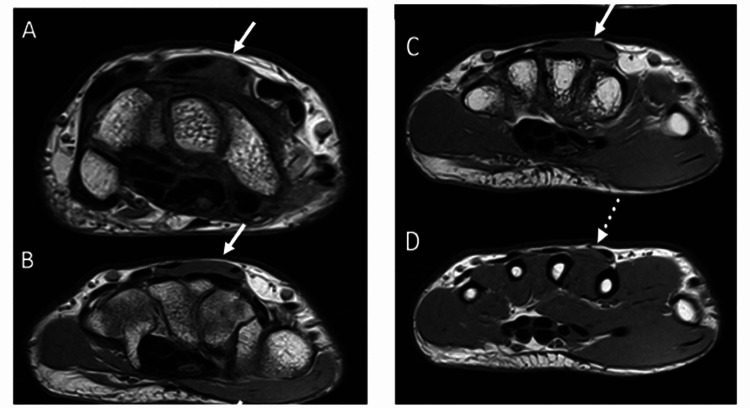
MRI hand There is an accessory muscle shown in images A, B, C, and D (white arrows) between the 2nd and 3rd extensor compartments of the dorsum of the wrist, consistent with the extensor digitorum brevis manus muscle. Triangular FibroCartilage Complex is Intact with normal morphology with no abnormal signal intensity. Scapho-Lunate and Lunotriquetral Ligament are Grossly intact. No scapholunate dissociation. Radiocarpal joint unremarkable. Distal radioulnar joint unremarkable. Midcarpal carpal joints are Grossly intact and have no erosive changes. Carpometacarpal joints are Grossly intact and have no definite erosive changes. Carpal bones are Maintained. Extensor and Flexor tendons are intact. Median Nerve is unremarkable. The ulnar nerve is unremarkable.

The patient was then referred to the radiology department for MRI. MRI of the right hand was obtained with gadolinium administration. There is an accessory muscle between the 2nd and 3rd extensor compartments of the dorsum of the wrist, consistent with the extensor digitorum brevis manus muscle. The ligaments, carpal bones, and joints were unremarkable (Figure 2). The patient was treated conservatively with rest, heat, ice, and therapeutic exercise and he was able to resume his daily life activities with no difficulties.

## Discussion

Extensor digitorum brevis manus (EDBM) originates from the extensor surface of the hand/wrist, the most common origin described is either the dorsal metacarpal surface, the distal end of the radius, or the proximal portion of the radiocarpal ligament [9-10]. The muscle is innervated by the posterior interosseous nerve [9-10]. The insertion of EDBM is of importance in the case of EDBM because of its relation with extensor indicis proprius (EIP), both might have a common insertion which is useful in classifying this muscle. According to Ogura et al., who have classified the muscle according to origin and insertion, there are three main types: type I where EIP is absent and only EDBM is present in which tendon is inserted onto the dorsal aponeurosis of the index finger. In type II, both EIP and EDBM had a common insertion on the index finger. In type III, both have a separate insertion, EIP inserted on the index finger and EDBM inserted on the middle finger, with or without an accessory EIP to the middle finger [10].

The action of EDBM was examined in most literature, which if stimulated deviates the proximal phalanx to the side on which it is inserted in relation to the extensor digitorum communis [11]. Gama C described an examination technique for patients who will benefit from surgery, which is resistance against finger extension, if it does induce pain then most likely the patient will benefit from surgery [11]. 

EDBM can be diagnosed by electromyography or by MRI like in our case. If the diagnosis was still in question, surgical exploration can be utilized [2]. 

The usual presentation of EDBM is a painless lump on the dorsum of the hand, however, many patients present with pain related to EDBM associated with repetitive use typically on flexion. Pain is explained by the constriction of the muscle belly by the extensor retinaculum, also termed the fourth compartment [11-12].

The recommended treatment in most literature was conservative unless the patient presented with pain. If a patient presents with pain, the initial management is with rest, heat, ice, bracing, and/or therapeutic exercise [13]. Wendel and Cole described a case that presented with pain and managed conservatively with Botulinum toxins. In their case, the pain was relieved for 1.5 years rather than three months which is the usual duration of action of Botulinum toxin [11]. Surgical treatment was also described for cases presenting with pain, such as extensor retinaculum release or resection of EDBE [10-13].

## Conclusions

In conclusion, we hereby present a case of EDBM that presented as a painful palpable mass on the dorsum of the hand. The diagnosis of EDBM can be misleading and easily overlooked, and can be confused with a ganglion cyst. Therefore, proper history taking and examination skills and choosing proper diagnostic modality such as MRI imaging are all necessary to properly diagnose EDBM.
